# Isolated Mitochondria State after Myocardial Ischemia-Reperfusion Injury and Cardioprotection: Analysis by Flow Cytometry

**DOI:** 10.3390/life13030707

**Published:** 2023-03-06

**Authors:** Claire Crola Da Silva, Delphine Baetz, Marie Védère, Mégane Lo-Grasso, Mariam Wehbi, Christophe Chouabe, Gabriel Bidaux, René Ferrera

**Affiliations:** University of Lyon, CARMEN Laboratory, INSERM, INRAE, Université Claude Bernard Lyon 1, 69500 Lyon, France

**Keywords:** mitochondria, ischemia reperfusion injury (IRI), postconditioning, flow cytometry

## Abstract

Rationale: Mitochondria are key organelles involved in cell survival and death during the acute phenomena of myocardial ischemia-reperfusion (i.e., myocardial infarction). To investigate the functions of isolated mitochondria such as calcium retention capacity, oxidative phosphorylation, and reactive oxygen species (ROS) production, already established methods are based on extramitochondrial measurements of the whole mitochondria population. Objective: The aim of this study was to develop a reliable and well-characterized method for multiparametric analysis of isolated single mitochondrion by flow cytometry (FC) in the context of myocardial infarction. The advantage of FC is the possibility to give a simultaneous analysis of morphological parameters (side and forward scatters: SSC and FSC) for each mitochondrion, combined with intramitochondrial measurements of several biological markers, such as ROS production or membrane potential (Δφm), using specific fluorescent probes. Methods and Results: For this study, a rat model of ischemia-reperfusion and a protective approach of post-conditioning using low reperfusion pressure was used. Thanks to the use of specific probes (NAO, MTR, TMRM, DilC1, and DHR123) combined with flow cytometry, we propose a method: (i) to identify mitochondrial populations of interest based on quality criteria (NAO/TMRM double staining); (ii) to monitor their morphological criteria, especially during swelling due to calcium overload; and (iii) to compare mitochondrial functions (membrane potential and ROS production) in different experimental groups. Applied to mitochondria from ischemic hearts, these measurements revealed that individual mitochondria are altered and that cardioprotection by low-pressure reperfusion reduces damage, as expected. Conclusions: Our results highlight FC as a reliable and sensitive method to investigate changes in mitochondrial functions and morphology in pathological conditions that disrupts their activity such as the case in ischemia-reperfusion. This methodological approach can be extended to other pathologies involving mitochondrial dysfunctions. Moreover, FC offers the possibility to work with very small amounts of isolated mitochondria, a factor that may limit the use of classical methods.

## 1. Introduction

Mitochondria are both the target and source of injury during myocardial ischemia and reperfusion [1]. During this pathophysiological condition, mitochondria become key players in cell death processes, involving a combination of deleterious signals such as increased oxidative stress by the production of reactive oxygen species (ROS), impaired calcium homeostasis, alteration of respiratory chain complexes, loss of mitochondrial membrane potential (Δφm), and opening of mitochondrial permeability transition pore (mPTP) inducing the release of apoptotic factors and ultimately cell death.

In order to discriminate the different molecular events occurring at the level of mitochondria, it was usual and relevant to isolate this organelle from tissue or organs. The most common techniques for the analysis of mitochondrial functions include spectrophotometry, spectrofluorometry, and oxygraphy tools [2,3]. However, these methods present three major limitations: (1) they require large amounts of isolated mitochondria, (2) they only provide measurements from the extramitochondrial medium (the events occurring in the mitochondrial matrix remain inaccessible with these techniques), and (3) the number of viable mitochondria analyzed is usually not evaluated. In addition, most of the analyses conducted on isolated mitochondria involve standardization methods based only on the quantification of the amount of mitochondrial protein and/or tissue weight. These methods do not take into account the number of functional or non-functional mitochondria present in the preparation. This can lead to misestimation of the mitochondrial functions assessed. For example, in the case of mitochondria extracted from tissues damaged by ischemia-reperfusion (IR), many of them are partially or totally destroyed and therefore non-functional but they are still counted when the protein content of the preparation is considered.

One of the fundamentals of flow cytometry (FC) is its ability to analyze the properties of individual particles [4]. FC uses the principles of light scattering, light excitation, and emission of fluorescent molecules to generate specific multi-parameter data (relative size, internal complexity, and relative fluorescence intensity) from plenty of biological samples such as particles, organelles, and cells with a size range from 0.2 µm to 150 µm. Fluorescence measurements, recorded at different wavelengths with different dyes, can provide quantitative and qualitative data.

Our study model is mitochondria isolated from rat hearts that have undergone ex vivo IR sequence. The purpose of this study was to develop a well-characterized protocol for the evaluation of mitochondria quality, measurement of the membrane potential, the ROS production, to characterize the mitochondrial swelling from a single mitochondrion. In addition, it examines the effect of postconditioning (POST) induced by low-pressure-reperfusion.

## 2. Materials and Methods

An expanded Methods section is available in the Methods supplement (where buffer compositions are described).

### 2.1. Dyes

MitoProbe™ DilC1 (1,1′,3,3,3′,3′-hexamethylindodicarbo-cyanine iodide), TetraMethylRhodamine Methyl ester (TMRM), FCCP (carbonylcyanide p-trifluoromethoxyphenylhydrazone), Nonyl acridine orange (NAO), MitoTracker^®^ Deep Red (MTR), Dihydrorhodamine 123 (DHR 123) were purchased from Life Technologies. Ascorbic acid, Rotenone, Antimycin A, Succinate, Hydrogen peroxide, ADP (adenosine diphosphate) were purchased from Sigma-Aldrich-France.

### 2.2. Surgical Procedure

The investigation conformed to the *Guide for the Care and Use of Laboratory Animals* published by the US National Institute of Health (NIH Publication No. 85-23, revised 1996).

With respect to regulations, our laboratory is authorized on behalf of the Direction of Veterinary Services. The experiments on rats were approved by the Ethical Committee of Claude Bernard University of Lyon (Comité en Expérimentation Animale, CEEA) with a subsequent validation by the national ethics committee with the following reference: 

Ref. CEEA: 2022-28v3.

Male Wistar rats (350–450 g) were placed in chamber containing sevoflurane for anesthesia induction. After the loss of consciousness, maintenance of anesthesia was conducted by using a breathing mask with sevoflurane (mac 4–5). Analgesia was performed with α_2_ agonist xylazine (10 mg/kg). General deep anesthesia was controlled and Heparin (200 IU/kg) was injected into the femoral vein. Euthanasia was performed by heart harvesting: the heart was excised and quickly harvested and arrested in ice-cold Krebs solution. The heart was subsequently perfused on the Langendorff apparatus using Krebs–Henseleit bicarbonate buffer (containing in mmol/L: glucose 11.0, NaCl 118.5, KCl 4.75, MgSO_4_ 1.19, KH_2_PO_4_ 1.18, NaHCO_3_ 25.0, CaCl_2_ 1.4) at pH 7.4. The buffer was equilibrated with 5% CO_2_ / 95% O_2_ bubbling at 37°C.

A balloon, connected to a pressure transducer, was introduced into the left ventricle to assess cardiac function, and the heart was paced at a constant rate of 300 beats/min, as previously described [2].

### 2.3. Ex Vivo Ischemia-Reperfusion

Three experimental groups were considered in this study (*n* = 6 per group):

- SHAM: This group of hearts underwent no ischemia and were immediately reperfused after harvesting, at normal pressure (i.e., 100 cm H_2_O) for the whole duration of the experiment (i.e., 60 min);
- IR group (ischemia-reperfusion): The hearts were submitted to 40 min of global ischemia at 37 °C followed by 60 min of reperfusion at normal pressure;
- POST group (postconditioning by low-pressure reperfusion). The method and its validation were previously described [2,5]. The myocardium was submitted to 40 min of global ischemia at 37 °C and was reperfused at low pressure (70 cm H_2_O) for the first 10 min then at normal pressure (100 cm H_2_O) for the remaining reperfusion time (50 min).

During the reperfusion time, functional recoveries were studied on the Langendorff apparatus. The heart rate (HR), the left ventricular systolic pressure (LVSP), and the left ventricular end-diastolic pressure (LVEDP) were measured using a latex balloon introduced into the left ventricle and expanded to exert a physiological end-diastolic pressure of 5 mmHg. The rate-pressure product [RPP = (LVSP − LVEDP) × HR], the maximum rate of rise of the LV pressure (dP/dt max), and the maximum isovolumetric rate of relaxation (dP/dt min) were calculated. Coronary flow was measured by periodic collections of the coronary effluent flow.

### 2.4. Preparation of Isolated Mitochondria

All operations were carried out at 4 °C. Myocardial left ventricles were placed in isolation-specific buffer A (containing in mmol/L: sucrose 70, mannitol 210, EGTA 1, Tris/HCl 50, pH 7.4). Tissue was finely minced with scissors and then homogenized in the same buffer, using a Kontes tissue grinder. Homogenate was centrifuged at 1300× *g* for 3 min. The supernatant was poured through cheesecloth and centrifuged at 10,000× *g* for 10 min. Mitochondrial pellet was resuspended in isolation-specific buffer B (same concentrations as buffer A except for EGTA reduced to 0.1 mM). Mitochondria were then centrifuged at 6800× *g* for 10 min and stored as pellets on ice prior to subsequent analysis. The mitochondrial protein amount was determined using Bradford Assay by spectrophotometry. We used, for each experiment, 10µg of mitochondrial protein.

### 2.5. Mitochondrial Oxygen Consumption

Oxygen consumption in freshly isolated mitochondria was measured at 25 °C with a Clark-type large-diameter Orbisphere oxygen electrode-controlled chamber (Oroboros Oxygraph, Paar, Graz, Austria). Mitochondria (250 µg proteins) were incubated in specific buffer C (containing in mmol/L: KCl 80, 4-morpholinepropanesulfonic acid 50, EGTA 1, KH_2_PO_4_ 5, and 1 mg/mL defatted BSA, pH 7.4). Pyruvate, malate, and glutamate (3 mmol/L for each compound) were used as substrates for electron donation to complex I. State 3 (200 µmol/L Adenosine Diphosphate: ADP addition), state 4 (ADP limited), and respiratory control index (RCI = state 3/state 4) were determined.

### 2.6. Dye Staining of Mitochondria

After isolation of mitochondria, 10 µg of mitochondria were resuspended in 1 mL of buffer C.

The different dyes were added at a suitable concentration and incubated for 10 min at room temperature away from light. Then, mitochondria were washed by adding 2 mL of buffer C. Mitochondria were pelleted by centrifugation (3000× *g* rpm, 10 min). A volume of 500 µL of buffer C was added for the acquisition by FC.

### 2.7. Calcium-Dependent Swelling of Mitochondria

Mitochondria from sham hearts were exposed to 40 nmol calcium pulses every minute. Changes in size and granularity were monitored using the variation of side and forward scatters (SSC and FSC) in time-dependent manner. Data acquisition was performed every minute after calcium loading. The results were represented in histogram plots, where the variation in SSC and FSC were represented by the peak shift of the population.

### 2.8. FC Analysis

FC analysis of isolated heart mitochondria was performed with a FACS Calibur equipped with a 488-nm argon laser and a 635-nm red diode laser (Becton Dickinson, San Jose, CA, USA). Data were analyzed using the CellQuest software. Firstly, mitochondria were identified based on SSC and FSC scatter plots. However, since scatters may vary from one cytometer to another or from one user to another (Figure S1A), our gating strategy for the quality criterion of the mitochondrial population studied is based on the double NAO/TMRM staining with a requirement of 90% minimum of double-positive mitochondria (Figure S1B).

Then, 100,000 double-positive events per sample were recorded. Each fluorescent probe is characterized by its excitation/emission (ex/em) wavelengths. The values for the dyes used in this study were: NAO (ex: 495 nm, em: 519 nm), MTR (ex: 644 nm, em: 665 nm), DilC1 (ex: 638 nm, em: 658 nm), TMRM (ex: 549 nm, em: 573 nm), DHR (ex: 507 nm, em: 529 nm).

### 2.9. Statistical Analysis

Statistical comparisons were performed using one-way ANOVA, followed, if significant, by Bonferroni post hoc analysis for multiple comparisons. Values are expressed in mean +/− SEM. A *p* value < 0.05 was considered statistically significant.

## 3. Results

Since the fluorescent probes are initially developed for cellular applications, working concentrations and loading protocols should be tailored to suit isolated mitochondria, especially to avoid toxicity.

Thus, as a preamble, in order to determine the optimal use concentration of each probe, and to verify the non-toxicity of these ones on the metabolism, we measured the respiratory control index (RCI) of mitochondria in different concentrations. The RCI is an essential test for assessing mitochondrial viability. It represents the capacity of the mitochondria to use the substrates to produce ATP (oxidative phosphorylation). For the subsequent experiments, we selected a working solution for each dye with a minimal concentration able to give a signal without toxic effect.

In a second step, we investigated, using FC, the effect of myocardial ischemia-reperfusion on mitochondrial dysfunction.

FIRST STEP: validation of the use of the different probes on isolated mitochondria for FC analysis.

### 3.1. Identification of Mitochondria by NAO and MTR Staining Combined with the Analysis of FSC and SCC (Figure 1)

Two probes were used to identify isolated mitochondria. NAO is well retained in the mitochondria. It has been previously reported to bind to cardiolipins, unique fatty acids in the inner mitochondrial membrane [6]. MTR is a cell-permeant probe that contains a mildly thiol-reactive chloromethyl moiety for labeling mitochondria.

**Figure 1:**
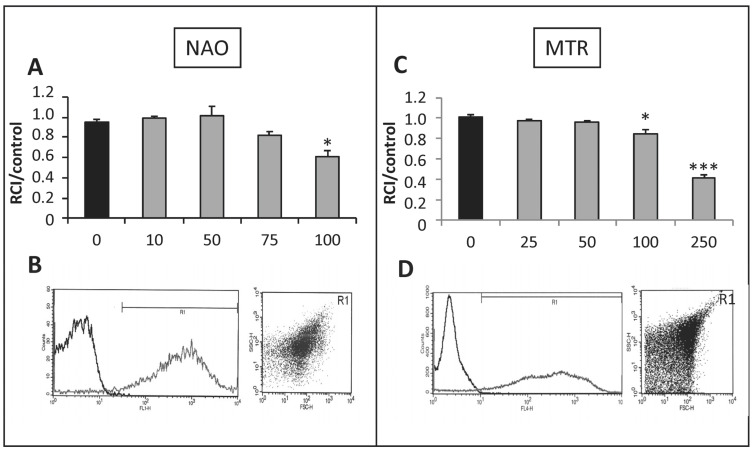
NAO and MTR as specific markers of mitochondria. The probe toxicity was determined by the RCI measurement in isolated mitochondria from rat hearts according to a range of concentrations from 10 to 100 nmol/L for NAO (**A**) and from 25 to 250 nmol/L for MTR (**C**). Loading of the isolated mitochondria with the optimal working concentrations (50 nmol/L for NAO and 25 nmol/L for MTR) enabled to properly gate the population of interest (**B**,**D**). Quality of mitochondria preparation was evaluated by combination of NAO staining with FSC/SSC dot plot. * *p* < 0.05 vs. 0 nmol/L. *** *p* < 0.001 vs. 0 nmol/L (*n* = 3 per group).

NAO had no significant effect on mitochondrial RCI up to 75 nmol/L but RCI was significantly decreased at 100 nmol/L (Figure 1A). Thus, we choose to use NAO at 50 nmol/L in the following experiments. MTR induced partial inhibition of mitochondrial RCI above 50 nmol/L (Figure 1C). It was used at 25 nmol/L for subsequent experiments.

Here, we validated the use of these two probes for the identification of isolated mitochondria: NAO staining (Figure 1B) and MTR staining (Figure 1D).

### 3.2. Analysis of Mitochondrial Swelling in Time-Lapse-Dependent Manner by External Calcium Pulses (Figure 2)

**Figure 2:**
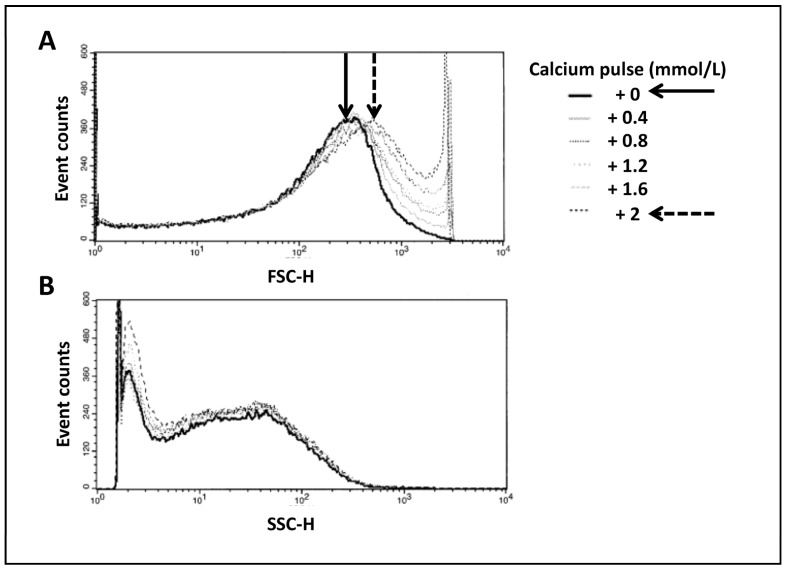
Swelling of mitochondria in time-lapse-dependent manner. Correlated measurements of FSC (**A**) and SSC (**B**) reflect the changes in mitochondrial morphology. Histograms show the variation in side or forward scatters per number of events. This figure illustrates an example of the results obtained from 6 different mitochondria preparations.

Changes in the volume of the mitochondrial matrix have an impact on mitochondrial function. Indeed, it has been previously shown [7] that small variations have a stimulating effect on the electron transfer chain and oxidative phosphorylation, whereas excessive swelling is associated with deleterious mechanisms for mitochondrial function and integrity, leading to cell death. We performed successive calcium pulses by the addition of CaCl_2_ to the mitochondrial suspension in order to induce a progressive swelling of mitochondria. Monitoring of FSC and SSC enabled us to follow changes in mitochondrial morphology. Proportionally to extracellular calcium concentration, we observed an increase in mitochondrial size as evidenced by a gradual increase in FSC (Figure 2A) correlated with a progressive decrease in SSC (Figure 2B), probably corresponding to the reorganization of the intramitochondrial matrix. Black and dashed arrows in Figure 2 denote this change.

This result pointed out the extreme sensibility of the FC, enabling us to distinguish very small variations in mitochondrial morphology.

### 3.3. Validation of DilC1 and TMRM Use to Evaluate Δφm in Isolated Mitochondria (Figure 3)

We tested the ability of two different probes to measure Δφm on SHAM mitochondria: DILC1 and TMRM.

**Figure 3:**
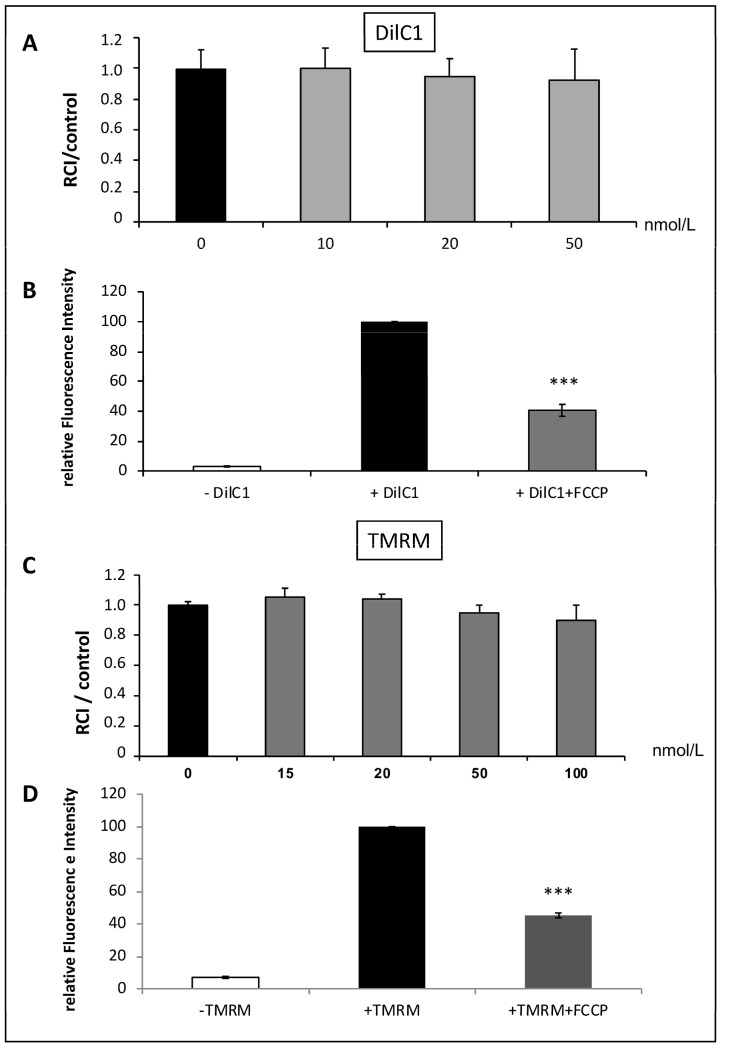
Validation of DilC1 use to measure Δφm in isolated mitochondria. The probe toxicity was determined by the RCI measurement in isolated mitochondria from rat hearts according to a range of concentrations from 10 to 50 nmol/L for DilC1 and from 15 to 100 nmol/L for TMRM (**A**,**C**). As expected, addition of the uncoupling agent FCCP at 10 µmol/L induced a decrease in DilC1 and TMRM fluorescence intensities (**B**,**D**), validating the ability of these probes to measure Δφm in our model. *** *p* < 0.001 vs. + DilC1 alone and vs. + TMRM alone (*n* = 3 per group).

DilC1 accumulates primarily in mitochondria with active Δφm and allows reliable measurement of Δφm on isolated cardiomyocytes [8]. After loading mitochondria with DilC1, we detected the population that had taken up the dye as compared to unstained mitochondria. In the concentration range tested (from 10 to 50 nmol/L), DilC1 did not modify the RCI of mitochondria (Figure 3A). For subsequent experiments, mitochondria were loaded with minimal concentration tested, 10 nmol/L.

The same experiments were conducted with the reference probe, TMRM. Similar results were obtained, with no toxic effect up to 50 nmol/L (Figure 3C) but provoked a slight but not significant decrease in RCI above this concentration. According to these results, TMRM was used at 20 nmol/L.

To validate the accuracy of the two probes used, we added 10 µmol/L of FCCP, the uncoupling agent, to the SHAM mitochondria. We observed a significant decrease in relative fluorescence intensity correlating with a loss of Δφm with both, DilC1 and TMRM (Figure 3B,D).

This step allowed us to validate the use of these two probes, in the upcoming experiments, to measure Δφm on isolated mitochondria in the different experimental groups.

### 3.4. Validation of DHR123 Use to Measure Intramitochondrial ROS Generation (Figure 4)

ROS formation was quantified using the conversion of dihydrorhodamine 123 (DHR 123) into Rhodamine 123 (Rh 123). DHR 123 is oxidized by intramitochondrial ROS to the cationic, membrane-impermeable fluorescent dye Rh123, which accumulates in mitochondria due to their negative inside environment. Thus, the fluorescence of “intramitochondrially” formed and trapped Rh 123 reflects mitochondrial ROS formation [9,10].

**Figure 4:**
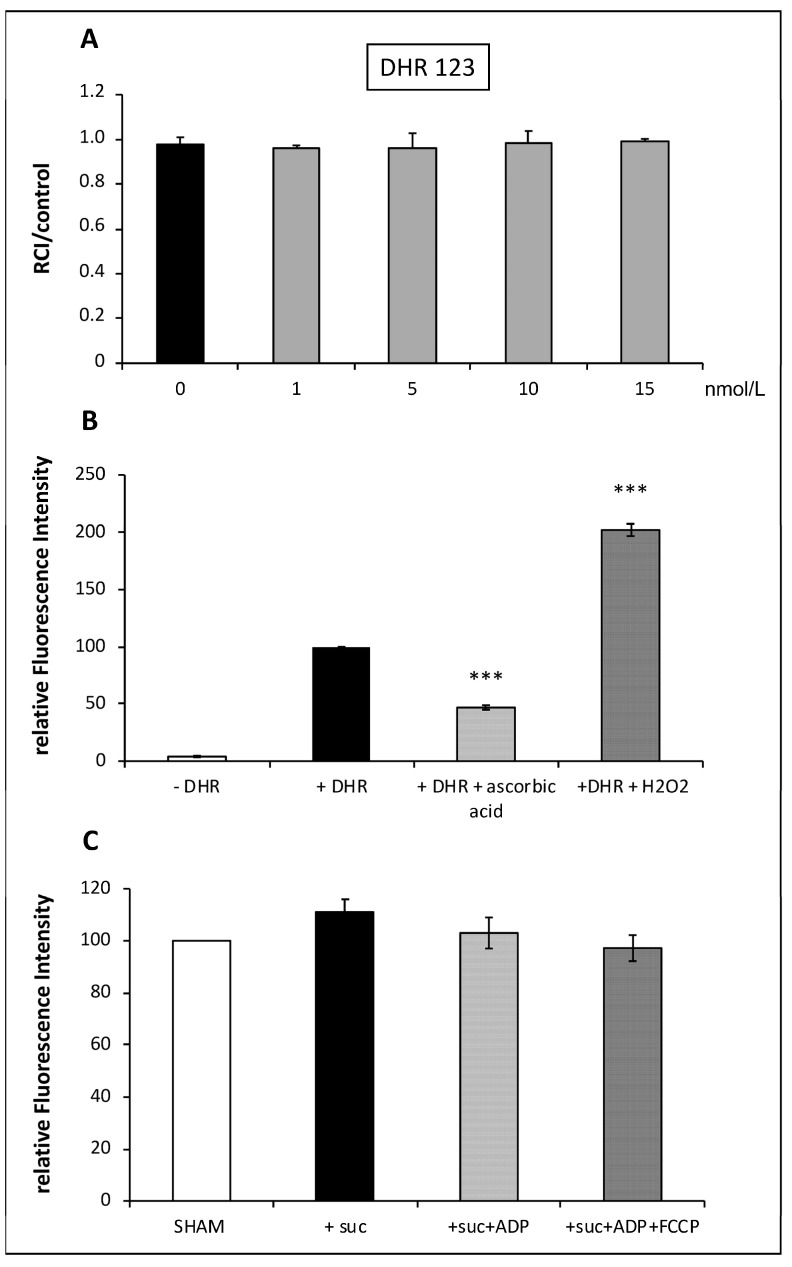
Validation of DHR 123 used to measure intramitochondrial ROS production. The toxicity was evaluated by RCI from 1 to 15 nmol/L (**A**). The ROS scavenger: ascorbic acid and hydrogen peroxide (H_2_O_2_) were used to validate the ability of DHR 123 to detect intramitochondrial ROS production (**B**). Addition of the uncoupling agent, FCCP, to succinate and ADP, enabled confirmation that the fluorescence of the probe was not dependent on the Δφm (**C**). *** *p* < 0.001 vs. + DHR alone (*n* = 4 per group).

DHR 123 staining up to 15 nmol/L did not alter the RCI (Figure 4A). In order to validate this probe, we added a ROS scavenger (ascorbic acid) or H_2_O_2_ into the mitochondrial preparation. As expected, after ascorbic acid addition, the fluorescence of Rh123 was decreased by about 50% when compared to the control, whereas the incubation with H_2_O_2_ was responsible for a doubling in the relative fluorescence intensity (Figure 4B). Rh 123 was described in the literature to be dependent on the Δφm [11]. To verify whether DHR 123 could serve to evaluate the intramitochondrial ROS production independently of the Δφm, the fluorescence emitted by the mitochondria loaded with the probe was determined after incubation with ADP and FCCP, which depolarized the mitochondria as seen in the DilC1 and TMRM experiments (Figure 3). The loss of Δφm induced by ADP and FCCP did not significantly affect Rh 123 fluorescence (Figure 4C). Thus, in our model, these results validated that the Rh 123 fluorescence, the product of DHR 123 conversion, only reflects intramitochondrial ROS production independently of the Δφm modification. In the subsequent experiments and according to these results, DHR 123 was loaded into mitochondria at 10 nmol/L.

SECOND STEP: Evaluation of the mitochondrial functions by FC after IR and postconditioning protocols

### 3.5. Ischemia Reperfusion Decreased Cardiac Functionality and Δφm, Which Were Partially Maintained by Postconditioning (Table 1 and Figure 5)

After harvesting, rat hearts were immediately reperfused (SHAM group), subjected to a sequence of 40 min warm ischemia followed by 60 min of reperfusion (IR group), or postconditioned by low-pressure reperfusion (POST group). At the end of the experiment, in sham hearts, the rate pressure product (RPP), our index of functionality, averaged 26,721 ± 164 mmHg/min at 60 min of reperfusion whereas it decreased sharply to 4765 ± 508 mmHg/min after the ischemic insult on IR hearts. Postconditioning significantly improved heart functional recovery with RPP averaging 10,163 ± 932 mmHg/min (*p* < 0.01 between IR and POST groups). Other functional parameters (LVDP, dp/dt max and min) showed similar improvement in the POST group as compared with the IR group (*p* < 0.001))—see Table 1.

**Table 1:** Functional recovery of isolated hearts, assessed at reperfusion.

Groups	RPP 60′ (mmHg/ min)	LVDP (mmHg)	HR (Beats/min)	dp/dt max (mmHg/ sec)	dp/dt min (mmHg/ sec)	CF (mL/min /mg of Dry Weight)
Sham	26,721 ± 164	97 ± 4	303 ± 2	1815 ± 72	1492 ± 63	94 ± 10
IR	4765 ± 508	8.33 ± 1	296 ± 16	136 ± 20	96 ± 18	52 ± 4
POST	10,163 ± 932 (**)	24.17 ± 1 (***)	305 ± 2	418 ± 17 (***)	325 ± 16 (***)	55 ± 5

Abbreviations: POST for postconditioning, RPP for rate pressure product, LVDP for left ventricular developed pressure, HR for heart rate, dP/dt max for maximum rate of rise of the LV pressure, dP/dt min for maximum isovolumetric rate of relaxation, and CF for mean coronary flow. *n* = 6 hearts/group. ** p < 0.01 and *** p < 0.001 differences between IR and POST.

**Figure 5:**
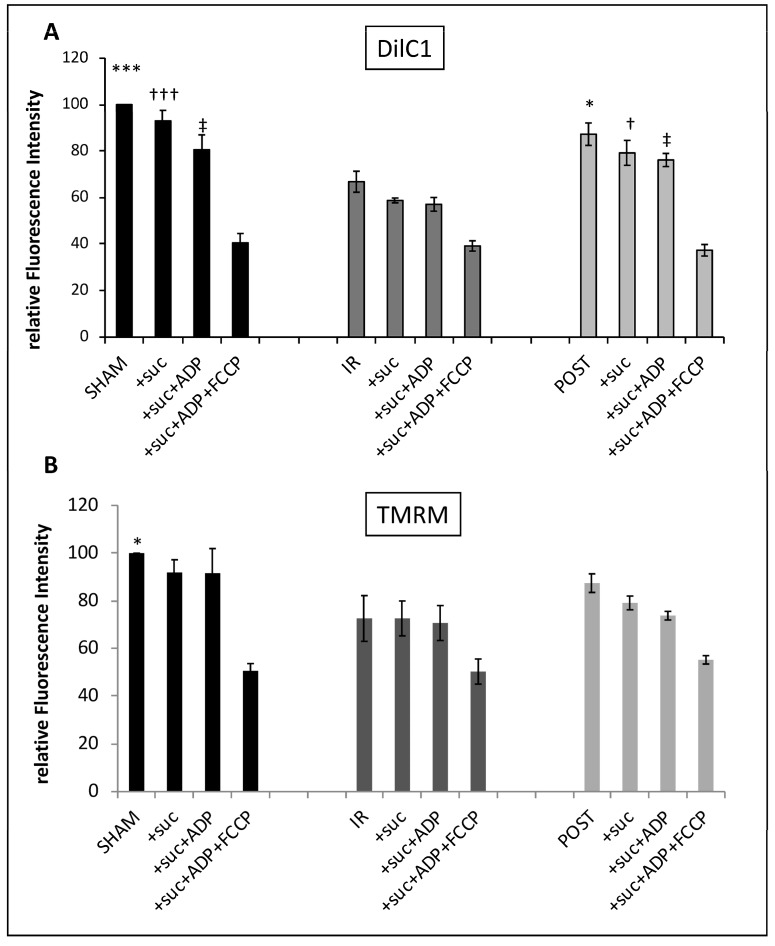
The Δφm is partially maintained after postconditioning. The Δφm was measured by incubating mitochondria from each group with DilC1 at 10 nmol/L (**A**) or TMRM at 20 nmol/L (**B**) after stimulation by succinate (3.75 µmol/L), ADP (6.25 µmol/L), and FCCP (10 µmol/L) successively in the same experimental tube. Results were expressed in relative fluorescence intensity. *^,†,‡^ *p* < 0.05 vs. IR and ***^,†††^ *p* < 0.001 vs. IR (*n* = 6 per group).

The mitochondria extracted from these three groups of hearts allowed us to analyze, using FC, mitochondrial membrane potential after IR with or without postconditioning, in parallel to the functional results. DilC1 staining revealed a slight depolarization after the addition of succinate and ADP in SHAM mitochondria. When we compared the different experimental conditions (basal, +suc, and +suc + ADP), we observed a significant loss of Δφm (40%) after IR when compared to SHAM (Figure 5A). Mitochondria isolated from postconditioned hearts displayed an intermediate profile with a 20% loss of Δφm as compared to SHAM mitochondria and a significant recovery when compared to the IR group. The addition of FCCP in each group provoked a significant decrease in Δφm where it reached a similar value whatever the group considered. A similar trend was obtained with TMRM (Figure 5B) but the differences between the IR and the POST group were not significant.

Thus, it appears that IR significantly altered the maintenance of Δφm and that was partially reversed by POST. Our results showed that DilC1 seems more sensitive than TMRM to reveal the modifications of Δφm in our experimental conditions.

### 3.6. Production of Intramitochondrial ROS Was Increased in IR Mitochondria and Partially Abolished by Postconditioning (Figure 6)

**Figure 6:**
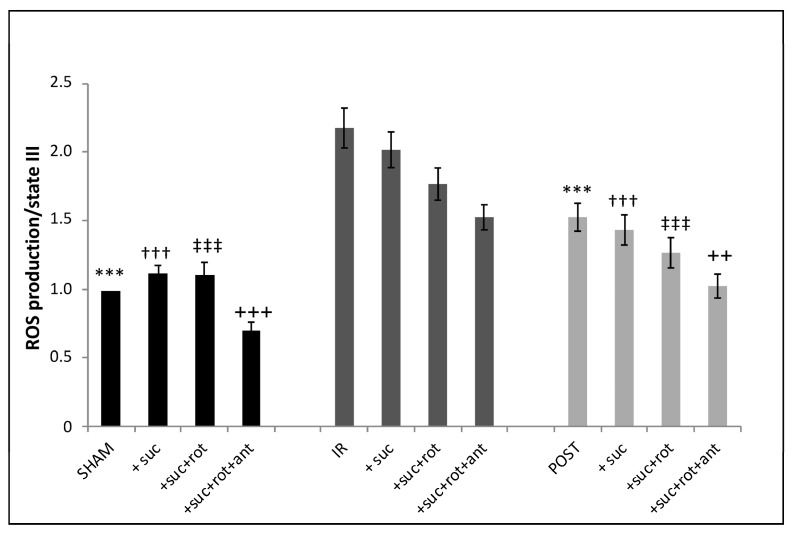
ROS production is increased by ischemia-reperfusion, which is partially limited by postconditioning. ROS production is detected in mitochondria after labeling with DHR at 10 nmol/L in the three experimental groups after stimulation by succinate (3.75 µmol/L), rotenone (6.25 µmol/L), and antimycin A (1 µmol/L successively in the same experimental tube). ***^,†††,‡‡‡,+++^ *p* < 0.001 vs. IR and ^++^ *p* < 0.01 vs. IR (*n* = 6 per group).

In SHAM mitochondria, the addition of the complex II substrate of the mitochondrial respiratory chain (succinate) provoked a significant increase (13.2%) in the fluorescence intensity as compared to the basal level of ROS. The addition of the complex I inhibitor, rotenone, which blocks the reverse transport of electrons [12], had no additional effect on ROS production as compared to the addition of succinate alone. However, the combined inhibition of complex I and complex III (addition of rotenone + antimycin A) led to a significant decrease (30%) in fluorescence intensity reflecting a decrease in ROS production. After IR, ROS production was significantly increased when compared to the SHAM group in the four different conditions (basal, +succinate, +succinate + rotenone, and +succinate + rotenone + antimycin). However, in the IR group, when the respiratory chain was stimulated by succinate, we were unable to show any additional increase in ROS production, unlike what was observed in the SHAM group. Although, as in the SHAM group, the addition of the inhibitors of complex I and III decreased ROS production compared to the basal IR condition. After postconditioning, overall, the ROS production showed the same profile as the one obtained in the IR group but with a general decrease in intensity. Thus, as expected, IR is associated with a significant ROS generation by mitochondria, which was partially blunted by POST (Figure 6).

## 4. Discussion

To summarize, in this present study, we propose the use of FC as an effective tool in the analysis of isolated mitochondrial functions after myocardial infarction. To our knowledge, no study to date has investigated the isolated single mitochondrion by flow cytometry in the context of myocardial ischemia-reperfusion and cardioprotection induced by low-pressure reperfusion.

Flow cytometry (FC) is a well-established method to assess mitochondrial function within whole cells but few studies mention its use to investigate mitochondrial function in isolated organelles preparation. Petit et al. were the first to use FC to detect isolated mitochondria in suspension initially from potatoes [13]. Since then, few studies used FC to analyze single mitochondrion generally extracted from muscle, liver, or brain [14,15,16]. Yet, the analysis of every single mitochondrion can reveal and quantify characteristics that are difficult to observe with traditional analysis techniques. For example, the existence of subpopulations or heterogeneities is difficult to observe on whole-cell preparations with a spectrofluorometer. The other advantage is the multiparametric approach to assess diverse mitochondrial functions such as the membrane potential, the analysis of the permeability of the transition pore, or the mitochondrial survival after cell suffering.

As far as possible, we deliberately chose two probes per parameter tested in order to validate the robustness of the analysis of isolated mitochondria by flow cytometry.

We chose to use NAO for its ability to bind to cardiolipins in the inner membrane of mitochondria. However, previous studies have shown that the binding of NAO to mitochondrial cardiolipins could be Δφm-dependent [6] or non-Δφm-dependent [17]. This dependence does not really affect our analysis since we used NAO in order to highlight our population of interest. In our study, NAO was only used for the selection of the mitochondria-containing population and thus for morphological analysis. The mitotracker deep red was also used in our population of interest gating strategy. The choice between NAO and MTR was made according to the other probes used in our panel. Concerning the measurement of Δφm, our data validated the use of DilC1 in isolated mitochondria. We tested DilC1 in comparison with TMRM, which is most commonly used to measure Δφm. The data were similar but the staining with DilC1 ensured more reproducibility and more sensitivity as compared with TMRM measurements in isolated mitochondria (Figure 3). This is probably due to a technical limitation of the cytometer used. Indeed, the lasers available on the FACS Calibur (488 nm and 633 nm) did not match the optimal excitation of TMRM (549 nm). Thus, we could not obtain the maximum emission of the probe as opposed to DilC1, which is excitable at 633 nm.

The basal level of DilC1 fluorescence intensity was significantly decreased in the IR group as compared to SHAM. Treatment of the heart by POST partially maintained the Δφm. The addition of succinate did not cause any significant variation as compared with the basal level (*p* > 0.05), whereas the addition of ADP, which induces a metabolic transition to state 3, provoked a transient depolarization (*p* < 0.05) in all groups, as expected, corresponding to the ATP synthesis [18]. Our results confirm the POST-induced mitochondrial protection against reperfusion injury, partly by maintaining the Δφm. It is related to a better mitochondrial functional integrity, in parallel with a better cardiac functional recovery.

For the recording of mitochondrial ROS production, it was not possible to validate the measure with two different probes; we only used DHR 123. Indeed, we have tested several fluorescent probes to detect ROS such as Mitosox red, H_2_DCFDA, or DAF-FM diacetate but none of them were suitable for direct use in isolated mitochondria either because they were toxic to mitochondria or because the probe needs intracellular processing to fluoresce, which was not possible in our conditions.

The presence of ROS in mitochondria was determined with DHR123 in three conditions after stimulation by succinate followed by successive addition of rotenone and antimycin A in the SHAM group and allowed us to verify that ROS production is dependent on mitochondrial membrane potential and the complexes integrity. Global ROS production was significantly increased in the IR group compared to the SHAM group whereas POST induced a smaller increase. These data highlight the variation in intramitochondrial ROS production during IR and postconditioning. FC revealed also that ROS generated by mitochondria were inside the matrix. To become deleterious for the cell, we can suppose that unidentified ROS transporters would exist on the inner mitochondrial membrane allowing the expulsion of ROS into the cytosol.

It is well known that significant mitochondrial swelling is most often associated with impaired mitochondrial function. We were able to show that successive CaCl2 pulses on mitochondria resulted in intramitochondrial reorganization (SSC) and size changes (FSC). Unfortunately, we did not see any significant modifications in SSC and FSC after IR and POST compared to the SHAM group. It would have been very interesting to compare the answer to calcium pulses in the different experimental groups.

## 5. Limitations, Strengths, and Perspectives

The strengths of FC include providing a multiparametric analysis combining morphological information with fluorescent qualitative measurements from a very low amount of mitochondria, which cannot be performed with classical methods such as oxygraphy and spectrofluorometry. Moreover, FC is suitable to evaluate mitochondria from various origins with the need for very low amounts of biological material: we tested up to 1 µg/mL of isolated mitochondria from rat, mouse, and rabbit hearts, which represents more than a hundred times less than what is needed when using conventional methods. The preparation of isolated mitochondria from tissues or cells is dependent on several environmental factors as well as the experimental procedure. We provide good quality control of mitochondrial preparation with the double staining of mitochondria marker combined with a membrane potential probe as illustrated here by the double staining NAO/TMRM. An additional benefit of the FC is the use of fluorescent probes that allow access to intramitochondrial information, unlike usual methods of mitochondria analysis.

As evoked previously, the major limitation of the use of FC in isolated mitochondria is the fact that the tools developed for flow cytometry are mostly dedicated to the cells.

Our results also open up a new perspective. It is well known that ischemic stress affects the global population of mitochondria but the effect on individual organelles is poorly documented. FC made it possible to observe it and revealed that every single mitochondrion was affected by the ischemic stress and participated in a global effect. It seems that mitochondria retain the traces of ischemic suffering. We can thus suppose that the isolated mitochondria, released by the suffering cells and found in the circulation after, for example, infarction, keep a functional trace that could perhaps constitute a good biomarker of the state of viability or even predict the future of the ischemic organ. The FC would then be a precious, original, and rapid tool that can be extended to other pathologies.

## 6. Conclusions

In conclusion, this work demonstrates that FC could be a reliable tool to investigate the morphology and intramitochondrial functions from very small amounts of identified mitochondria isolated from ischemic or non-ischemic myocardium.

## Data Availability

The data presented in this study are available on request from the corresponding author. The data are not publicly available due to privacy.

## Supplementary Materials

The following supporting information can be downloaded at: https://www.mdpi.com/article/10.3390/life13030707/s1, Figure S1A: Representative dot plots of mitochondria scatters. Figure S1B: Representative dot plots of the gating strategy based on the double staining with NAO and TMRM probes.

## Abbreviations

DILC1	1:1′,3,3,3′,3′-hexamethylindodicarbo-cyanine iodide
DHR 123	Dihydrorhodamine 123
FC	Flow Cytometry
FCCP	carbonylcyanide p-trifluoromethoxyphenylhydrazone
FSC	Forward Scatter
IR	Ischemia-Reperfusion
LPR	Low Pressure Reperfusion
mPTP	mitochondrial permeability transition pore
MTR	Mitotracker Deep Red
NAO	Nonyl Acridine Orange
POST	Postconditioning
RCI	Respiratory Control Index
Rh 123	Rhodamine 123
ROS	reactive oxygen species
RPP	Rate Pressure Product
SSC	Side SCatter
TMRM	TetraMethylRhodamine Methyl Ester
Δφm	Mitochondrial membrane potential
