# Supplementary material for: Isolated Mitochondria State after Myocardial Ischemia-Reperfusion Injury and Cardioprotection: Analysis by Flow Cytometry
Source: Life (Basel). 2023 Mar 6;13(3):707. doi: 10.3390/life13030707 (PMC10053810; doi:10.3390/life13030707)
Supplement: Supplementary file 1 [file life-13-00707-s001.zip › life-2225821-supplementary.pdf]

Isolated mitochondria state after myocardial ischemia-reperfusion injury and cardioprotection. Analysis by flow cytometry.

Claire CROLA DA SILVA; Delphine BAETZ ; Marie VÉDÈRE; Mégane LO-GRASSO; Mariam Wehbi; Christophe CHOUABE; Gabriel BIDAUX and René FERRERA

University of Lyon; CARMEN Laboratory; INSERM; INRAE; Université Claude Bernard Lyon 1; 69500 Lyon, France
*Corresponding author: E-mail : rene.ferrera@univ-lyon1.fr; Phone: (33) 04 78 77 72 42.

SUPPLEMENTAL DATA

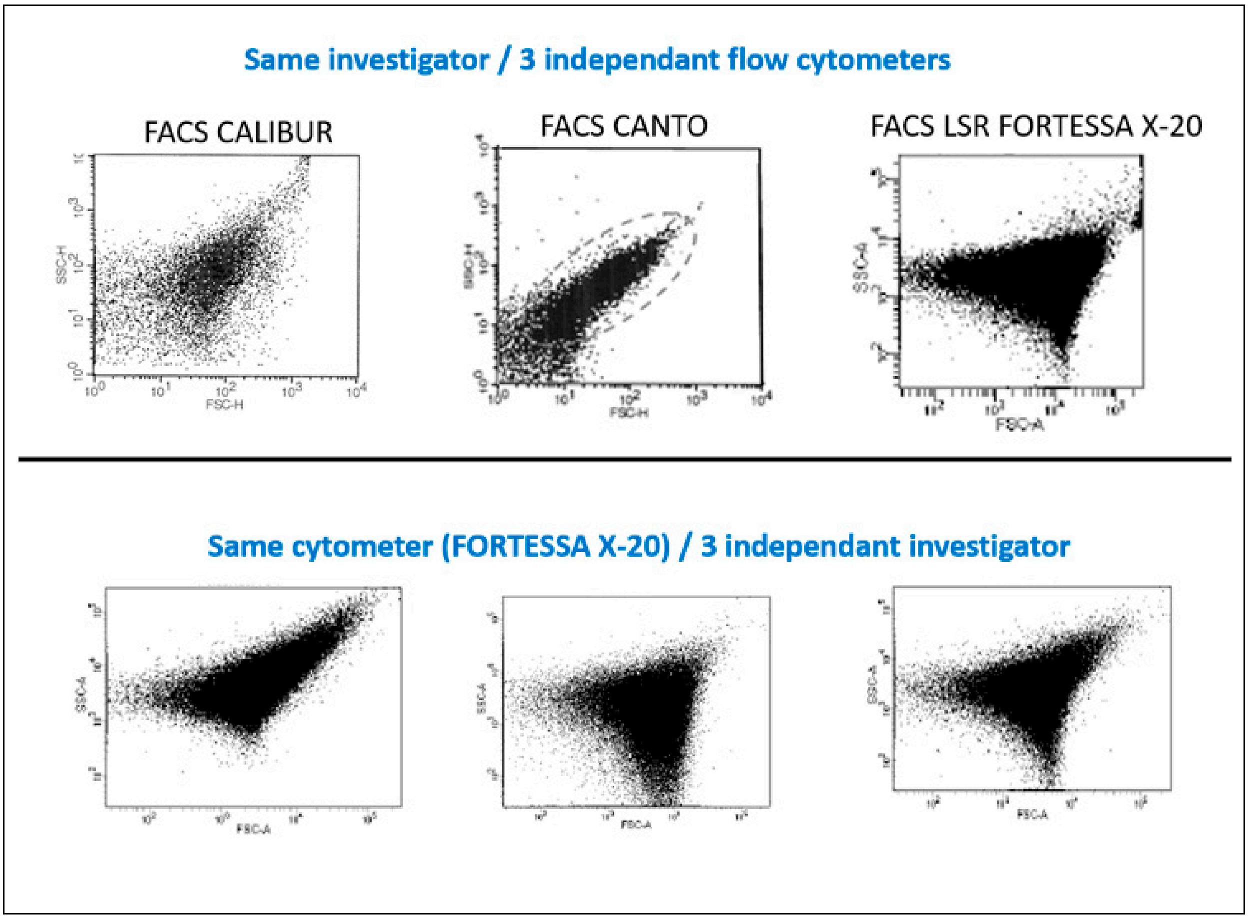

Figure S1A: Representative dot plots of mitochondria scatters. The variability of scatters may vary according to the type of flow cytometer used for investigation as well as according to the investigator in charge of mitochondria isolation.

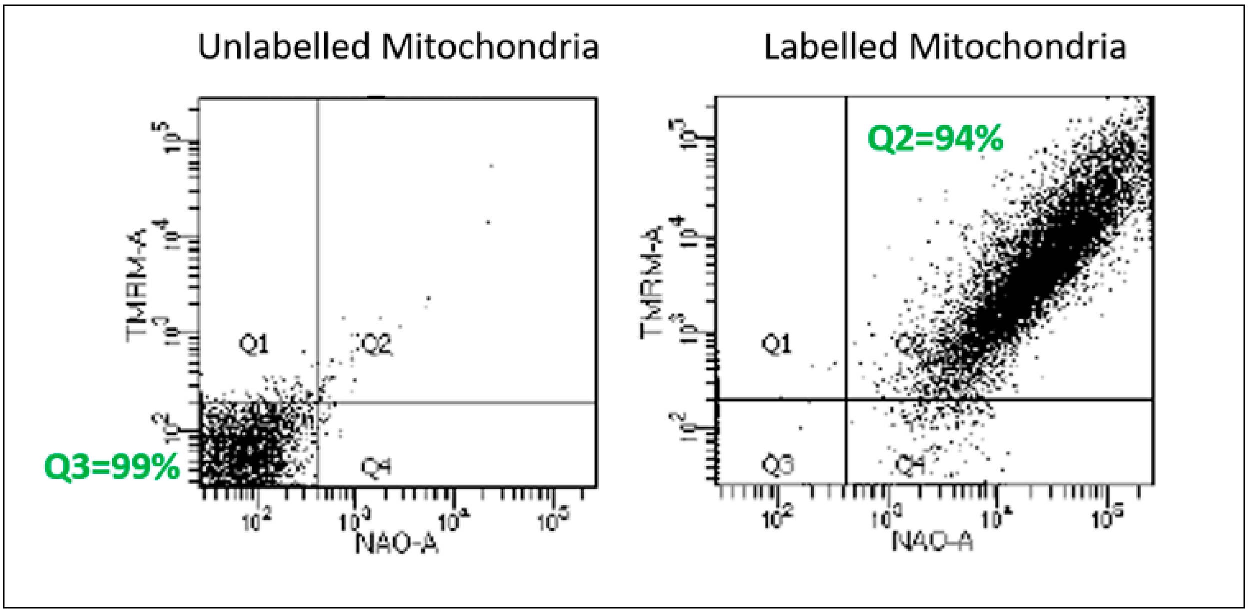

Figure S1B: Representative dot plots of the gating strategy based on the double staining with NAO and TMRM probes. We expected a minimum of 90% of double positive mitochondria as represented here by Q2.
